# Current smoking is associated with extracranial carotid atherosclerotic stenosis but not with intracranial large artery disease

**DOI:** 10.1186/s12883-017-0873-7

**Published:** 2017-06-26

**Authors:** Ruijun Ji, Yuesong Pan, Hongyi Yan, Runhua Zhang, Gaifen Liu, Penglian Wang, Yilong Wang, Hao Li, Xingquan Zhao, Yongjun Wang, On behalf of The Chinese Intracranial Atherosclerosis (CICAS) Study investigators

**Affiliations:** 1 Department of Neurology, Tiantan Comprehensive Stroke Center, Tiantan Hospital, Capital Medical University, Beijing, China; 2 China National Clinical Research Center for Neurological Diseases, Beijing, China; 3 Center of Stroke, Beijing Institute for Brain Disorders, Beijing, China; 4 Beijing Key Laboratory of Translational Medicine for Cerebrovascular Disease, Beijing, China; 5 Beijing Key Laboratory of Brain Function Reconstruction, Beijing, China; 6 Tiantan Comprehensive Stroke Center, Beijing Tiantan Hospital, Capital Medical University, No.6 Tiantanxili, Dongcheng District, Beijing, 100050 China

**Keywords:** Stroke, Smoking, Extracranial atherosclerotic stenosis, Intracranial atherosclerotic stenosis, Association

## Abstract

**Background:**

Accumulating evidence has shown that cigarette smoking is an important risk factor for ischemic stroke. However, it is not clear about the potential mechanisms through which cigarette smoking affects stroke risk. In the study, we aimed to investigate the relationship between cigarette smoking and the occurrence of extracranial (ECAS) and intracranial atherosclerotic stenosis (ICAS).

**Methods:**

We analyzed patients enrolled in the Chinese intracranial atherosclerosis (CICAS), which was a prospective, multicenter, hospital-based cohort study. Smoking status was classified into never, former and current smoking. For those patients with current smoking, data on time duration (year) and extent (the number of cigarette smoked per day) was recorded and pack year of smoking was calculated. ICAS was evaluated with 3-dimentional time-of-flight MRA and ECAS was evaluated with cervical ultrasonography or contrast-enhanced MRA. Multivariable Logistic regression was performed to identify the association between smoking status and the occurrence of ECAS and ICAS.

**Results:**

A total of 2656 patients (92.7%) of acute ischemic stroke and 208 (7.3%) of transient ischemic attack were analyzed. The mean age was 61.9 ± 11.2 and 67.8% were male. There were 141 (4.9%) patients had only ECAS, 1074 (37.5%) had only ICAS, and 261 (9.1%) had both ECAS and ICAS. Current smoking was significantly associated with the occurrence of ECAS (adjusted OR = 1.47, 95% CI = 1.09–1.99, *P* < 0.01). In addition, with 1 year of smoking increment, the risk of ECAS increased by 1.1% (adjusted OR = 1.011; 95% CI = 1.003–1.019; *P* = 0.005); with one cigarette smoked per day increment, the risk of ECAS increased by 1.0% (adjusted OR = 1.010; 95% CI = 1.001–1.020; *P* = 0.03); and with one pack year of smoking increment, the risk of ECAS increased by 0.7% (adjusted OR = 1.007; 95% CI = 1.002–1.012; *P* < 0.01). However, no significant association was found between smoking status and the occurrence of ICAS.

**Conclusion:**

A dose–response relationship was identified between cigarette smoking and the occurrence of ECAS, but not ICAS. Further studies on molecular mechanisms were warranted.

**Electronic supplementary material:**

The online version of this article (doi:10.1186/s12883-017-0873-7) contains supplementary material, which is available to authorized users.

## Background

Cigarette smoking is responsible for approximately five million deaths every year and there are still an estimated 1.1 billion smokers worldwide [1–3]. Accumulating evidence has shown that cigarette smoking is an important risk factor for first ischemic stroke [4]. In contrast to extensive data on association between cigarette smoking and risk for first stroke, data on its association with recurrent stroke are sparse [5]. In the Cardiovascular Health Study, cigarette smoking was associated with a 2-fold increased risk for stroke recurrence in the elderly [6].

The mechanisms through which cigarette smoking affects stroke risk have been a topic of interest over the last several years. Although it is not fully understood, cigarette smoking likely contributes to increased stroke risk through both short-term effect on thrombus generation in atherosclerotic arteries and long-term effect related to the development of atherosclerotic stenosis [7]. Clinically, atherosclerotic stenosis of cervicocephalic arteries could be classified into extracranial (ECAS) and intracranial atherosclerotic stenosis (ICAS). ECAS is the most common vascular lesion found in stroke patients of White. In contrast, ICAS is found more common among stroke patients of Asian, Black, and Hispanic ancestry [8]. Thus far, limited is known about the potential role of cigarette smoking in the development of ECAS and ICAS.

In the present study, we aimed to clarify the relationship between time duration, the number of cigarette smoked per day and pack years of smoking and the occurrence of ECAS and ICAS.

## Methods

### Study population

This study was based on the Chinese intracranial atherosclerosis (CICAS) study [9], which was a prospective, multicenter, hospital-based cohort study aimed at clarifying the prevalence and risk of recurrent stroke in patients with intracranial large artery occlusive disease. Briefly, 22 general hospitals covering a wide geographic area in China participated in the CICAS. The inclusion criteria of CICAS study were: (1) age between 18 and 80 years; (2) hospitalized with a primary diagnosis of acute ischemic stroke (AIS) or transient ischemic attack (TIA) according to the World Health Organization criteria; (3) stroke confirmed by head computerized tomography (CT) and/or brain magnetic resonance imaging (MRI); (4) time from symptom onset to hospitalization < 7 days. The CICAS study excluded those patients who were: (1) with known source for cardioembolism; (2) disabled before admission (modified Rankin Scale [mRS] score > 2); (3) physically or subjectively unable to comply with MRI examination; (4) clinically unstable or required close monitoring or were moribund. The CICAS study had been approved by the institutional review board (IRB) at each participating hospital. All participants provided written informed consent.

### Study variables

#### Clinical characteristics

In the CICAS network, a standardized case report form (CRF) was used for data collection. For the present study, the following candidate variables were analyzed: (1) demographics (age and gender); (2) stroke risk factors: hypertension (defined by a history of hypertension, or being treated with an antihypertensive agent before admission, or diagnosed at discharge), diabetes mellitus (defined by a history of diabetes mellitus, or being treated for diabetes mellitus or glycosylated hemoglobin ≥ 7%, or diagnosis at discharge), dyslipidemia (defined as a self-reported history, current use of lipid-lowering medications, total cholesterol level ≥ 5.7 mmol/L, or triglyceride level ≥ 1.7 mmol/L on admission), family history of stroke, history of cerebral ischemia (including a history of ischemic stroke and TIA), history of hemorrhagic stroke (including intracerebral hemorrhage and subarachnoid hemorrhage), heart disease (defined as a history of myocardial infarction, angina pectoris, and congestive heart failure), smoking status, and heavy drinking (drinking >2 units per day on average for men or >1 unit per day on average for women); (3) comorbidities: chronic obstructive pulmonary disease (COPD), hepatic cirrhosis, peptic ulcer or previous gastrointestinal bleeding (GIB), arthritis, Alzheimer’s disease/dementia, cancer and peripheral angiopathy; (4) Admission systolic (SBP) and diastolic blood pressure (DBP) (mmHg); (5) Admission National Institutes of Health Stroke Scale (NIHSS) score; (6) admission blood tests: fasting blood glucose (mmol/L), triglyceride (TG) (mmol/L), cholesterol (TC) (mmol/L), high density lipoprotein (HDL) (mmol/L) and low density lipoprotein (LDL) (mmol/L).

#### Smoking status

In the CICAS study, smoking status was classified into never, former and current smoking. Current smoking was defined as a patient who had smoked continuously for 6 months with at least one cigarette per day. Former smoking was defined as a patient who was used to smoke but not satisfied the criteria of current smoking. For those patients with current smoking, data on time duration (year) and extent (the number of cigarette smoked per day) was recorded and pack year of smoking was calculated. The pack year is a unit for measuring the amount a person has smoked over a long period of time. It is calculated by multiplying the number of packs of cigarettes smoked per day by the number of years the person has smoked.

#### Vascular imaging of ECAS and ICAS

In the CICAS study, all patients underwent conventional MRI on a 3.0-T or 1.5-T MR scanner, including sequences of 3-D time-of-flight MRA, T_2_/T_1_-weighted imaging, fluid-attenuated inversion recovery sequences (FLAIR), and diffusion-weighted imaging (DWI). All MRI and MRA images were read centrally by two readers who were blinded to patients’ clinical information. Disagreement involving more than 10% degree of stenosis was resolved by an appointed senior reader who decided the final classification. The following arterial segments were assessed: 1) bilateral extracranial and intracranial internal carotid artery (ICA), anterior cerebral artery (ACA) A1/A2, middle cerebral artery (MCA) M1/M2, posterior cerebral artery (PCA) P1/P2 and basilar artery (BA). The degree of intracranial stenosis on MRA was calculated using the method of the WASID study [10] and was classified into four groups: <50% or no stenosis, 50% to 69%, 70% to 99%, and occlusion. The extracranial internal carotid artery was evaluated with ultrasonography according to the published diagnostic criteria [11] or by contrast-enhanced MRA. In the present study, significant stenosis was defined as a more than 50% atherosclerotic stenosis or occlusion of the large intracranial and/or extracranial arteries. Based on the presence and location of cerebral atherosclerotic stenosis, patients were classified into four groups: 1) without ECAS or ICAS; 2) with only ECAS; 3) with only ICAS; and 4) with both ECAS and ICAS.

### Statistical analysis

Continuous variables were summarized with mean and standard deviation (SD) or median and interquartile range (IQR); categorical variables were summarized as proportions. In univariate analysis, Chi-square or Fisher exact test was used to compare categorical variables and one way analysis of variance or Kruskal-Wallis test was employed to compare continuous variables. In multivariable analysis, Logistic regression was performed to assess association between smoking status (never, former and current smoking) and the occurrence of ECAS, ICAS and specific location of ICAS (intracranial ICA, MCA, ACA, PCA and BA). Logistic regression adjusted for demographics (age and gender), stroke risk factors, comorbidities, admission NIHSS score, admission SBP and DBP, and admission blood tests. All tests were 2-tailed and statistical significance was determined at α level of 0.05. Statistical analysis was performed using SAS 9.1 (SAS Institute, Cary, NC) and SPSS 17.0 (SPSS Inc., Chicago, IL).

## Results

### Patient characteristics

Patient characteristics are shown in Table 1. A total of 2864 patients (2656 [92.7%] of AIS and 208 [7.3%] of TIA) were enrolled in the CICAS network. The mean age was 61.9 ± 11.2 and 67.8% were male. There were 1568 (54.8%) patients who never smoked, 247 (8.6%) with former smoking and 1049 (36.6%) with current smoking. A total number of 1476 (51.5%) patients had either ECAS or ICAS. Compared with patients without ECAS or ICAS, those developed ECAS or ICAS were more common for older age, male gender, presence of diabetes mellitus, hypertension, dyslipidemia and cerebral ischemia, family history of stroke, current or former smoking, higher NIHSS score, higher blood glucose, and lower blood HDL (Table 1).

**Table 1:** Baseline characteristics

Variables	Overall (*n* = 2864)	No ECAS or ICAS (*n* = 1388)	Only ECAS (*n* = 141)	Only ICAS (*n* = 1074)	ECAS + ICAS (*n* = 261)	*P* value
Age (Mean ± SD)	61.9 ± 11.2	61.2 ± 11.2	64.7 ± 10.4	62.1 ± 11.5	63.6 ± 10.1	<0.001
Gender (male)	1944(67.8)	938(67.6)	110(78.0)	696(64.8)	200(76.6)	<0.001
Stroke risk factors						
Diabetes mellitus	991(34.6)	414(29.3)	50(35.5)	418(38.9)	109(41.8)	<0.001
Hypertension	2238(78.1)	1056(76.1)	105(74.5)	866(80.6)	211(80.8)	0.02
Hyperlipidemia	2173(75.9)	1042(75.1)	119(84.4)	807(75.1)	205(78.5)	0.05
Family history of stroke	296(10.3)	122(8.8)	16(11.4)	112(10.4)	46(17.6)	<0.001
History of cerebral ischemia	2018(70.5)	930(67.0)	117(83.0)	772(71.9)	199(76.3)	<0.001
History of hemorrhagic stroke	54(1.9)	29(2.1)	3(2.1)	19(1.8)	3(1.2)	0.73
Coronary heart disease	228(8.0)	102(7.4)	17(12.1)	88(8.2)	21(8.1)	0.30
Heavy drinking	142(5.0)	66(4.8)	9(6.4)	51(4.8)	16(6.1)	0.66
Smoking status						<0.001
Never smoker	1568(54.8)	774(55.8)	53(37.6)	623 (58.0)	118 (45.2)	
Former smoker	247(8.6)	118(8.5)	17(12.1)	84 (7.8)	28 (10.7)	
Current smoker	1049(36.6)	496(35.7)	71(50.4)	367 (34.2)	115 (40.1)	
Comorbidities						
COPD	12(0.4)	8(0.6)	1(0.7)	2(0.2)	1(0.4)	0.47
Hepatic cirrhosis	7(0.2)	1(0.1)	0(0.0)	5(0.5)	1(0.4)	0.22
Peptic ulcer or previous GIB	80(2.8)	43(3.1)	6(4.3)	23(2.1)	8(3.1)	0.34
Arthritis	46(1.6)	16(1.2)	3(2.1)	26(2.4)	1(0.4)	0.03
Dementia	19(0.7)	8(0.6)	1(0.7)	9(0.8)	1(0.4)	0.81
Cancer	48(1.7)	23(1.7)	5(3.6)	17(1.6)	3(1.2)	0.32
Peripheral angiopathy	21(0.7)	9(0.7)	1(0.7)	7(0.7)	4(1.5)	0.47
Index event						0.01
Transient ischemic attack	208(7.3)	106(7.6)	19(13.5)	62(5.8)	21(8.1)	
Acute ischemic stroke	2656(92.7)	1282(92.4)	122(86.5)	1012(94.2))	240(92.0)	
SBP at admission, mm Hg (median, IQR)	150(135–167)	150(136–165)	150(130–165)	150(135–170)	150(135–162)	0.31
DBP at admission, mm Hg (median, IQR)	88(80–95)	89(80–96)	85(80–90)	87(80–95)	85(80–95)	0.23
NIHSS at admission (median, IQR)	4(1–7)	3(1–5)	3(1–5)	4(2–8)	5(2–10)	<0.001
Fasting blood glucose, mmol/L (median, IQR)	5.4(4.8–6.8)	5.3(4.7–6.6)	5.3(4.8–6.2)	5.7(4.9–7.2)	5.8(4.8–7.1)	<0.001
TG, mmol/L (median, IQR)	1.5(1.1–2.1)	1.5(1.1–2.2)	1.6(1.1–2.1)	1.5(1.1–2.1)	1.4(1.1–2.0)	0.15
TC, mmol/L (median, IQR)	4.66(4.00–5.38)	4.67(4.06–5.40)	4.58(3.87–5.33)	4.68(3.98–5.38)	4.50(3.95–5.35)	0.15
HDL, mmol/L (median, IQR)	1.12(0.96–1.32)	1.15(0.98–1.36)	1.08(0.95–1.29)	1.09(0.95–1.29)	1.08(0.91–1.27)	<0.001
LDL, mmol/L (median, IQR)	2.82(2.27–3.45)	2.83(2.26–3.43)	2.74(2.28–3.40)	2.84(2.28–3.47)	2.82(2.25–3.49)	0.91

*Abbreviations*: *ICAS* indicates intracranial atherosclerotic stenosis, *ECAS* extracranial atherosclerotic stenosis, *SD* standard deviation, *COPD* chronic obstructive pulmonary disease, *GIB* gastrointestinal bleeding, *SBP* systolic blood pressure, *DBP* diastolic blood pressure, *IQR* interquartile range, *NIHSS* National Institutes of Health Stroke Scale, *TG* triglyceride, *TC* cholesterol, *HDL* high density lipoprotein, *LDL* low density lipoprotein

### Proportion of ECAS and ICAS

The proportions of ECAS and ICAS in the CICAS study are shown in Additional file 1: Table S1. A total number of 141 (4.9%) patients had only ECAS, 1074 (37.5%) had only ICAS, and 261 (9.1%) had both ECAS and ICAS. For those patients developing ICAS, the most frequent location was MCA stenosis (29.6%), then followed by PCA stenosis (18.6%), ACA stenosis (7.7%), BA stenosis (6.3%), and intracranial ICA (2.3%) (Additional file 1: Table S1).

### Association between smoking status and ECAS or ICAS

Association between smoking status and the occurrence of ECAS or ICAS is shown in Table 2. Compared with patients never smoked, those with current smoking were significantly associated with the occurrence of ECAS (adjusted OR = 1.47, 95% CI = 1.09–1.99, *P* < 0.01). Although there is a trend that former smoking was associated with ECAS (adjusted for age, gender, and stroke risk factors), it did not reach statistical significance after adjusting all potential confounders (adjusted OR = 1.42, 95% CI = 0.92–2.18, *P* = 0.11). When combining former and current smoking together, ever smoking was significantly associated with the occurrence of ECAS (adjusted OR = 1.46, 95% CI = 1.09–1.94, *P* < 0.01). However, no similar association was found between cigarette smoking and the occurrence of ICAS or specific location of ICAS (intracranial ICA, MCA, ACA, PCA and BA) (Table 2).

**Table 2:** Association between smoking status and ECAS and ICAS in all enrolled patients (*N* = 2864)

Arterial stenosis	Category	Unadjusted OR	*P* value	Adjusted OR_1_	*P* value	Adjusted OR_2_	*P* value	Adjusted OR_3_	*P* value
ECAS	Previous smoking vs. never	1.82(1.27–2.61)	0.001	1.57(1.07–2.30)	0.02	1.49(1.01–2.09)	0.04	1.42(0.92–2.18)	0.11
	Current smoking vs. never	1.76(1.41–2.20)	<0.001	1.82(1.39–2.38)	<0.001	1.74(1.33–2.27)	<0.001	1.47(1.09–1.99)	<0.01
	Smoking vs. never	1.77(1.43–2.19)	<0.001	1.76(1.36–2.26)	<0.001	1.67(1.30–2.16)	<0.001	1.46(1.09–1.94)	<0.01
ICAS	Previous smoking vs. never	0.93(0.71–1.21)	0.58	0.94(0.71–1.25)	0.66	0.91(0.69–1.22)	0.54	1.01(0.71–1.44)	0.95
	Current smoking vs. never	0.95(0.81–1.11)	0.51	1.01(0.84–1.22)	0.91	0.99(0.82–1.20)	0.93	0.92(0.73–1.15)	0.45
	Smoking vs. never	0.94(0.82–1.10)	0.45	1.00(0.83–1.19)	0.95	0.97(0.82–1.17)	0.77	0.93(0.75–1.16)	0.53
Intra-ICA	Previous smoking vs. never	1.61(0.96–2.72)	0.07	1.42(0.98–2.11)	0.21	1.39(0.80–2.42)	0.25	1.26(0.65–2.45)	0.50
	Current smoking vs. never	1.58(1.14–2.18)	0.005	1.44(0.82–2.47)	0.06	1.34(0.91–1.98)	0.14	1.13(0.71–1.79)	0.61
	Smoking vs. never	1.58(1.16–2.16)	0.003	1.43(0.99–2.07)	0.05	1.35(0.94–1.95)	0.11	1.15(0.74–1.79)	0.52
MCA	Previous smoking vs. never	1.01(0.75–1.36)	0.94	1.01(0.74–1.37)	0.97	0.99(0.73–1.36)	0.97	1.05(0.72–1.53)	0.79
	Current smoking vs. never	1.03(0.87–1.23)	0.70	1.02(0.83–1.25)	0.86	1.01(0.82–1.24)	0.94	0.89(0.70–1.14)	0.36
	Smoking vs. never	1.03(0.88–1.21)	0.73	1.02(0.84–1.23)	0.87	1.01(0.83–1.22)	0.96	0.92(0.73–1.16)	0.50
ACA	Previous smoking vs. never	1.01(0.62–1.65)	0.97	1.17(0.69–1.97)	0.56	1.12(0.66–1.89)	0.69	0.93(0.49–1.76)	0.83
	Current smoking vs. never	0.86(0.63–1.16)	0.31	1.09(0.76–1.56)	0.64	1.04(0.73–1.50)	0.82	1.00(0.65–1.54)	0.99
	Smoking vs. never	0.89(0.67–1.17)	0.39	1.11(0.79–1.55)	0.56	1.06(0.75–1.49)	0.74	0.99(0.66–1.48)	0.94
PCA	Previous smoking vs. never	1.16(0.84–1.60)	0.37	1.29(0.92–1.83)	0.14	1.24(0.88–1.76)	0.22	1.40(0.93–2.11)	0.10
	Current smoking vs. never	0.71(0.58–0.88)	0.001	0.91(0.71–1.16)	0.45	0.88(0.68–1.13)	0.32	0.92(0.68–1.23)	0.57
	Smoking vs. never	0.79(0.65–0.96)	0.01	0.99(0.79–1.25)	0.94	0.96(0.76–1.21)	0.72	1.02(0.77–1.34)	0.90
BA	Previous smoking vs. never	0.91(0.53–1.56)	0.73	0.90(0.51–1.60)	0.73	0.83(0.47–1.47)	0.52	0.79(0.40–1.53)	0.48
	Current smoking vs. never	0.71(0.51–1.00)	0.05	0.81(0.55–1.20)	0.30	0.81(0.54–1.20)	0.28	0.88(0.56–1.40)	0.59
	Smoking vs. never	0.75(0.55–1.02)	0.07	0.83(0.58–1.20)	0.33	0.81(0.56–1.17)	0.26	0.86(0.56–1.32)	0.48

*Abbreviations*: *ICAS* indicates intracranial atherosclerotic stenosis, *ECAS* extracranial atherosclerotic stenosis, *OR* odds ration, *Intra-ICA* intracranial internal carotid artery, *MCA* middle cerebral artery, *ACA* anterior cerebral artery, *PCA* posterior cerebral artery, *BA* basilar artery

OR_1_ adjusted for demographics (age and gender). OR_2_ adjusted for demographics (age and gender) and stroke risk factors (diabetes mellitus, hypertension, dyslipidemia, family history of stroke, history of cerebral ischemia, history of hemorrhagic stroke, heart disease). OR_3_ adjusted for all potential confounders including demographics, (age and gender), stroke risk factors (diabetes mellitus, hypertension, dyslipidemia, family history of stroke, history of cerebral ischemia, history of hemorrhagic stroke, heart disease), comorbidities (COPD, hepatic cirrhosis, peptic ulcer or previous GIB, arthritis, dementia, cancer and peripheral angiopathy), admission NIHSS, admission SBP and DBP, and admission blood tests (fasting blood glucose, TG, TC, HDL and LDL)

### Association between time duration of smoking and ECAS or ICAS

**Fig. 1:**
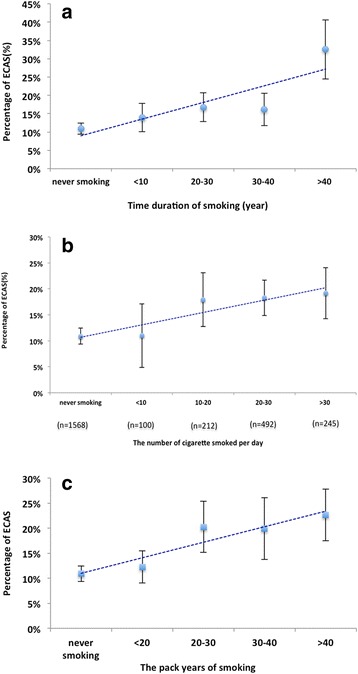
relationship between cigarette smoking the development of extracranial atherosclerotic stenosis (ECAS)**.** The risk of ECAS increased steadily with longer time duration (**a**), larger amount of cigarette smoked per day (**b**), and larger number of pack years (**c**) of smoking

Association between time duration of smoking and the occurrence of ECAS or ICAS was investigated in the subgroup of patients who were with never and current smoking (*n* = 2617). The risk of ECAS increased steadily with longer time duration of smoking (Fig. 1a). After adjusting for all potential confounders, significant association was found between time duration of smoking and the occurrence of ECAS. With 1 year of smoking increment, the risk of developing ECAS increased by 1.1% (adjusted OR = 1.011; 95% CI = 1.003–1.019; *P* = 0.005). However, no significant association was found between time duration of smoking and the occurrence of ICAS or specific location of ICAS (intracranial ICA, MCA, ACA, PCA and BA) (Additional file 1: Table S2).

### Association between extent of smoking and ECAS or ICAS

Association between extent of smoking and the occurrence of ECAS or ICAS was investigated in the subgroup of patients who were with never and current smoking (*n* = 2617). The risk of ECAS increased steadily with larger amount of cigarette smoked per day (Fig. 1b). After adjusting for all potential confounders, significant association was found between the number of cigarette smoked per day and the occurrence of ECAS. With one cigarette smoked per day increment, the risk of developing ECAS increased by 1.0% (adjusted OR = 1.010; 95% CI = 1.001–1.020; *P* = 0.03). However, no significant association was found between extent of smoking and the occurrence of ICAS or specific location of ICAS (intracranial ICA, MCA, ACA, PCA and BA) (Additional file 1: Table S3).

### Association between pack years of smoking and ECAS or ICAS

Association between pack years of smoking and the occurrence of ECAS or ICAS was investigated in the subgroup of patients who were with never and current smoking (*n* = 2617). The risk of ECAS increased steadily with larger number of pack years of smoking (Fig. 1c). After adjusting for all potential confounders, significant association was found between pack years of smoking and the occurrence of ECAS. With one pack year of smoking increment, the risk of developing ECAS increased by 0.7% (adjusted OR = 1.007; 95% CI = 1.002–1.012; *P* < 0.01). However, no significant association was found between pack years of smoking and the occurrence of ICAS or specific location of ICAS (intracranial ICA, MCA, ACA, PCA and BA) (Additional file 1: Table S4).

## Discussion

In the present study, we systematically investigated the relationship between cigarette smoking and the occurrence of ECAS and ICAS. It was found that cigarette smoking was significantly associated with the occurrence of ECAS. In addition, dose–response relationship between time duration, extent and pack years of cigarette smoking and the occurrence of ECAS was identified. However, no similar association was found between cigarette smoking and the occurrence of ICAS or specific location of ICAS (intracranial ICA, MCA, ACA, PCA, and BA).

The evidence linking cigarette smoking to stroke is extremely convincing and dose–response relationship has been well established [4, 5, 12–14]. However, the mechanisms through which cigarette smoking affects stroke risk are not well understood and have been a topic of interest over the last decades. Cigarette smoking likely contributes to increased stroke risk through both short-term effect on thrombus generation in atherosclerotic arteries and long-term effect on the development of atherosclerotic stenosis. Thus far, several studies have investigated the relationship between cigarette smoking and the occurrence of extracranial or intracranial atherosclerosis. To evaluate the prevalence and risk factors of asymptomatic carotid artery disease, Dr. Willeit and Kiechl analyzed a sample drawn from the community-based Study and found that pack years of smoking was the leading risk factor of carotid atherosclerosis in men [15]. In a cross-sectional study carried out in patients attending a lipid clinic, Dr. Baldassarre and his colleagues found that carotid intima-media thickness was significantly associated with pack years of smoking in both former and current smokers [14]. In the Atherosclerosis Risk in Communities (ARIC) Study, Dr. Howard and his colleagues found that exposure to cigarette smoking was associated with progression of atherosclerosis. Relative to never smokers, current cigarette smoking was associated with 50% increase and past smoking was associated with 25% increase in intimal-medial thickness of carotid artery over 3 years follow-up [16]. By using transcranial Doppler ultrasonography in patients who had at least one vascular risk factor of hypertension, diabetes, or hyperlipidemia, Dr. Wong and his colleagues found that age, hypertension, diabetes, and hyperlipidemia were associated with the occurrence of MCA stenosis; however, cigarette smoking was not significant [17]. Based on data from a consecutive series of subjects aged more than 40 years and without history of stroke, Dr. Bae and his colleagues found that age, hypertension, and diabetes were significantly associated with the occurrence of ICAS; however, no significant association was observed with regard to male gender, cigarette smoking and hyperlipidemia [18]. In a meta analysis including 15 Asian studies, Dr. Ding and his colleagues found that female or patients with metabolic syndrome were more likely to suffer from ICAS than ECAS; whereas, the smoker or patients with dyslipidemia were more likely to suffer from ECAS than ICAS [19]. Similar with these findings, our study verified that cigarette smoking was more associated with ECAS than ICAS. In addition, our study provided further evidence to indicate a dose–response relationship between cigarette smoking and the occurrence of ECAS. It was shown that with 1 year of smoking, one cigarette smoked per day, and one pack year of smoking increment, the risk of developing ECAS increased by 1.0, 1.1 and 0.7%, respectively. These numbers not only quantitatively showed the detrimental effect of cigarette smoking on cerebral atherosclerosis, but also suggested the potential effect of smoking cessation on stroke prevention. If we assumes an attributable risk of stroke as associated with ECAS of 0.4% per year [20] and the mean direct medical cost of ischemic stroke during acute hospitalization of 10,000 RMB (≈1548.25 US dollar with the exchange rate of 6.4589), taken in conjunction with the 350,000,000 smokers in China, then, if all Chinese stopped smoking, there would be 3,500,000 fewer ECAS and 14,000 fewer strokes every year. The cost benefits alone would be enormous, potentially saving 140,000,000 RMB (≈21,675,517.50 US dollar) per year spent on acute stroke care in China.

Although a clear association between smoking and health risk has long been established, the prevalence of smoking in our society remains alarmingly high. Smoking can be potentially reduced by population and individual-related measures. Smoke-free laws in certain areas have already been shown to be effective in reducing heart disease [21], declining in heart disease death [22], and improving health outcomes [23]. In addition, nowadays, smokers have more help than ever before to quit successfully [24, 25]. Given the clear evidence that intervention can lead to healthy change, clinicians, researchers, policy-maker, social workers, smokers and their relatives should be organized together to establish reliable, feasible, and cost-effective networks and pathways to create an environment in which smoking is unacceptable and to decrease the burden of smoking on health continuously.

Indeed, limited is known about the molecular mechanisms underlying the paradoxical relationship between cigarette smoking and the development of ECAS and ICAS. Previous studies have shown that cigarette smoke exposure had complex and extensive roles in vasomotor dysfunction, inflammation, oxidative stress and modification of lipids [26–28], which are integral components for initiation and progression of atherosclerosis. Due to sorts of difference in anatomical structure and local hemodynamics, these intermediate factors induced by cigarette smoking might have different role in the development of intracranial and extracranial atherosclerosis. Further studies on molecular mechanisms are warranted.

Our study has limitations that deserve mention. First, like all observational studies, we cannot rule out the possibility that additional variable (unmeasured confounders, e.g. medications used before hospitalization) might have some impact on ECAS and ICAS. Second, our study included only hospitalized patients and those patients died in emergency room, treated in outpatient clinics and asymptomatic patients were not included. In addition, our study required informed consent and selection bias was inevitable [29]. Third, in our study, intracranial artery stenosis was evaluated with 3-D time-of-flight MRA. Currently, digital subtract angiography (DSA) remains the gold standard for the diagnosis of arterial stenosis. Although MRA is noninvasive and more easily accessible compared with DSA [30], time-of-flight MRA is prone to artifacts because of flow abnormalities [31]. Finally, information on environmental tobacco smoke was not collected. Evidence has shown that exposure to environmental tobacco smoke is an established risk factor for heart disease [32, 33]. Meanwhile, we did not collect relevant information on the type of cigarette smoked by the subjects. With these kinds of information included in the study, we would have more convincing conclusion.

## Conclusion

We found a paradoxical dose–response relationship between cigarette smoking and the occurrence of ECAS and ICAS. Our study would encourage further studies to clarify molecular mechanisms underlying the different role of cigarette smoking in the development of ECAS and ICAS, as hopefully would pave ways to develop different acute care and preventive strategies for stroke patients with different smoking status.

## Additional file

Additional file 1: Table S1. Proportion of intracranial and extracranial atherosclerotic stenosis. Showed the proportion of intracranial and extracranial atherosclerotic stenosis (*n* = 2864). **Table S2.** Association between time duration of smoking and ECAS and ICAS in subgroup of patients with never and current smoking. Showed the association between time duration of smoking and ECAS and ICAS in subgroup of patients with never and current smoking (*n* = 2617). **Table S3.** Association between extent of smoking and ECAS and ICAS in subgroup of patients with never and current smoking. Showed the Association between extent of smoking and ECAS and ICAS in subgroup of patients with never and current smoking (*n* = 2617). (DOCX 46 kb)

## Abbreviations

ACA: Anterior cerebral artery
AIS: Acute ischemic stroke
BA: Basilar artery
CICAS study: the Chinese intracranial atherosclerosis (CICAS) study
COPD: Chronic obstructive pulmonary disease
CRF: Case Report Form
CT: Computerized tomography
DBP: Diastolic blood pressure
DSA: Digital subtract angiography
DWI: Diffusion-weighted imaging
ECAS: Extracranial atherosclerotic stenosis
FLAIR: Fluid-attenuated inversion recovery sequences
GIB: Gastrointestinal bleeding
HDL: High density lipoprotein
ICA: Internal carotid artery
ICAS: Intracranial atherosclerotic stenosis
IQR: Interquartile range
IRB: Institutional Review Board
LDL: Low density lipoprotein
MCA: Middle cerebral artery
MRI: Magnetic resonance imaging
mRS: modified Rankin Scale
NIHSS: National Institutes of Health Stroke Scale
PCA: Posterior cerebral artery
SBP: Systolic blood pressure
SD: Standard deviation
TC: Cholesterol
TG: Triglyceride
TIA: Transient ischemic attack
