# Supplementary material for: Current smoking is associated with extracranial carotid atherosclerotic stenosis but not with intracranial large artery disease
Source: BMC Neurol. 2017 Jun 26;17:120. doi: 10.1186/s12883-017-0873-7 (PMC5485653; doi:10.1186/s12883-017-0873-7)
Supplement: Additional file 1: Table S1. — Proportion of intracranial and extracranial atherosclerotic stenosis. Showed the proportion of intracranial and extracranial atherosclerotic stenosis (n = 2864). Table S2. Association between time duration of smoking and ECAS and ICAS in subgroup of patients with never and current smoking. Showed the association between time duration of smoking and ECAS and ICAS in subgroup of patients with never and current smoking (n = 2617). Table S3. Association between extent of smoking and ECAS and ICAS in subgroup of patients with never and current smoking. Showed the Association between extent of smoking and ECAS and ICAS in subgroup of patients with never and current smoking (n = 2617). (DOCX 46 kb) [file 12883_2017_873_MOESM1_ESM.docx]

**Supplementary materials**

Supplementary table 1. Proportion of intracranial and extracranial atherosclerotic stenosis

| Location of cervicocephalic arterial stenosis | Overall  (n=2864) |
| --- | --- |
| No ICAS or ECAS | 1388 (48.5%) |
| Only ECAS | 141 (4.9%) |
| Only ICAS | 1074 (37.5%) |
| MCA | 848 (29.6) |
| PCA | 523 (18.6) |
| ACA | 219 (7.7) |
| BA | 181 (6.3%) |
| Intra-ICA | 175 (6.1%) |
| ICAS+ECAS | 261 (9.1%) |

Abbreviations: ICAS indicates intracranial atherosclerotic stenosis; ECAS extracranial atherosclerotic stenosis.

**Supplementary table 2. Association between time duration of smoking and ECAS and ICAS in subgroup of patients with never and current smoking（n=2617）**

| Arterial stenosis | Category | Unadjusted OR | P value | Adjusted OR_1_ | P value | Adjusted OR_2_ | P value | Adjusted OR_3_ | P value |
| --- | --- | --- | --- | --- | --- | --- | --- | --- | --- |
| ECAS | Per 1 year increment | 1.022 (1.015-1.028) | <0.001 | 1.018 (1.011-1.025) | <0.001 | 1.017 (1.010-1.024) | <0.001 | 1.011 (1.003-1.019) | 0.005 |
| ICAS | Per 1 year increment | 1.002 (0.997-1.007) | 0.37 | 1.003 (0.998-1.009) | 0.20 | 1.003 (0.998-1.008) | 0.27 | 1.000 (0.994-1.006) | 0.96 |
| Intra-ICA | Per 1 year increment | 1.012 (1.003-1.022) | 0.007 | 1.009 (0.999-1.019) | 0.09 | 1.007 (0.996-1.017) | 0.20 | 1.003 (0.991-1.016) | 0.58 |
| MCA | Per 1 year increment | 1.001 (0.996-1.006) | 0.73 | 1.001 (0.995-1.006) | 0.83 | 1.001 (0.995-1.006) | 0.82 | 0.997 (0.990-1.004) | 0.35 |
| ACA | Per 1 year increment | 1.003 (0.994-1.011) | 0.51 | 1.008 (0.998-1.018) | 0.10 | 1.006 (0.997-1.016) | 0.19 | 1.004 (0.993-1.016) | 0.48 |
| PCA | Per 1 year increment | 0.998 (0.992-1.004) | 0.58 | 1.004 (0.997-1.011) | 0.23 | 1.003 (0.996-1.010) | 0.36 | 1.002 (0.994-1.010) | 0.57 |
| BA | Per 1 year increment | 0.993 (0.983-1.003) | 0.14 | 0.994 (0.983-1.005) | 0.27 | 0.994 (0.983-1.005) | 0.27 | 0.994 (0.982-1.006) | 0.32 |

Abbreviations: ICAS indicates intracranial atherosclerotic stenosis; ECAS extracranial atherosclerotic stenosis; OR odds ration; Intra-ICA intracranial internal carotid artery; MCA middle cerebral artery; ACA anterior cerebral artery; PCA posterior cerebral artery; BA basilar artery.

OR_1_ adjusted for demographics (age and gender). OR_2_ adjusted for demographics (age and gender) and stroke risk factors (diabetes mellitus, hypertension, dyslipidemia, family history of stroke, history of cerebral ischemia, history of hemorrhagic stroke, heart disease). OR_3_ adjusted for all potential confounders including demographics, (age and gender), stroke risk factors (diabetes mellitus, hypertension, dyslipidemia, family history of stroke, history of cerebral ischemia, history of hemorrhagic stroke, heart disease), comorbidities (COPD, hepatic cirrhosis, peptic ulcer or previous GIB, arthritis, dementia, cancer and peripheral angiopathy), admission NIHSS, admission SBP and DBP, and admission blood tests (fasting blood glucose, TG, TC, HDL and LDL).

**Supplementary table 3. Association between extent of smoking and ECAS and ICAS in subgroup of patients with never and current smoking（n=2617）**

| Arterial stenosis | Category | Unadjusted OR | P value | Adjusted OR_1_ | P value | Adjusted OR_2_ | P value | Adjusted OR_3_ | P value |
| --- | --- | --- | --- | --- | --- | --- | --- | --- | --- |
| ECAS | Per 1 cigarette per day increment | 1.017 (1.010-1.025) | <0.001 | 1.019 (1.010-1.027) | <0.001 | 1.018 (1.009-1.026) | <0.001 | 1.010 (1.001-1.020) | 0.03 |
| ICAS | Per 1 cigarette per day increment | 0.999 (0.993-1.004) | 0.63 | 1.001 (0.994-1.008) | 0.76 | 1.001 (0.994-1.007) | 0.81 | 0.998 (0.990-1.005) | 0.55 |
| Intra-ICA | Per 1 cigarette per day increment | 1.007 (0.996-1.019) | 0.20 | 1.003 (0.990-1.016) | 0.65 | 1.001 (0.988-1.014) | 0.89 | 1.000 (0.985-1.015) | 0.97 |
| MCA | Per 1 cigarette per day increment | 1.000 (0.994-1.007) | 0.99 | 0.999 (0.992-1.006) | 0.84 | 0.999 (0.992-1.006) | 0.81 | 0.997 (0.988-1.005) | 0.43 |
| ACA | Per 1 cigarette per day increment | 0.997 (0.986-1.009) | 0.65 | 1.005 (0.993-1.018) | 0.39 | 1.005 (0.992-1.017) | 0.47 | 1.006 (0.992-1.021) | 0.42 |
| PCA | Per 1 cigarette per day increment | 0.990 (0.981-0.998) | 0.01 | 0.998 (0.990-1.007) | 0.73 | 0.998 (0.989-1.007) | 0.67 | 0.998 (0.987-1.008) | 0.74 |
| BA | Per 1 cigarette per day increment | 0.986 (0.972-1.000) | 0.04 | 0.992 (0.977-1.007) | 0.27 | 0.992 (0.977-1.007) | 0.31 | 0.990 (0.973-1.007) | 0.25 |

Abbreviations: ICAS indicates intracranial atherosclerotic stenosis; ECAS extracranial atherosclerotic stenosis; OR odds ration; Intra-ICA intracranial internal carotid artery; MCA middle cerebral artery; ACA anterior cerebral artery; PCA posterior cerebral artery; BA basilar artery.

OR_1_ adjusted for demographics (age and gender). OR_2_ adjusted for demographics (age and gender) and stroke risk factors (diabetes mellitus, hypertension, dyslipidemia, family history of stroke, history of cerebral ischemia, history of hemorrhagic stroke, heart disease). OR_3_ adjusted for all potential confounders including demographics, (age and gender), stroke risk factors (diabetes mellitus, hypertension, dyslipidemia, family history of stroke, history of cerebral ischemia, history of hemorrhagic stroke, heart disease), comorbidities (COPD, hepatic cirrhosis, peptic ulcer or previous GIB, arthritis, dementia, cancer and peripheral angiopathy), admission NIHSS, admission SBP and DBP, and admission blood tests (fasting blood glucose, TG, TC, HDL and LDL).

**Supplementary table 4. Association between pack-years of smoking and ECAS and ICAS in subgroup of patients with never and current smoking（n=2617）**

| Arterial stenosis | Category | Unadjusted OR | P value | Adjusted OR_1_ | P value | Adjusted OR_2_ | P value | Adjusted OR_3_ | P value |
| --- | --- | --- | --- | --- | --- | --- | --- | --- | --- |
| ECAS | Per 1 pack-year increment | 1.013 (1.009-1.018) | <0.001 | 1.012 (1.007-1.016) | <0.001 | 1.011 (1.006-1.016) | <0.001 | 1.007 (1.002-1.012) | <0.01 |
| ICAS | Per 1 pack-year increment | 1.001 (0.997-1.004) | 0.65 | 1.002 (0.998-1.006) | 0.39 | 1.001 (0.997-1.005) | 0.50 | 1.000 (0.996-1.004) | 0.99 |
| Intra-ICA | Per 1 pack-year increment | 1.006 (1.000-1.013) | 0.06 | 1.004 (0.997-1.011) | 0.29 | 1.002 (0.995-1.010) | 0.53 | 1.001 (0.993-1.010) | 0.80 |
| MCA | Per 1 pack-year increment | 1.000 (0.996-1.004) | 0.97 | 1.000 (0.995-1.004) | 0.86 | 1.000 (0.995-1.004) | 0.86 | 0.998 (0.994-1.003) | 0.53 |
| ACA | Per 1 pack-year increment | 1.002 (0.996-1.009) | 0.44 | 1.006 (0.999-1.013) | 0.08 | 1.005 (0.998-1.012) | 0.16 | 1.005 (0.998-1.013) | 0.18 |
| PCA | Per 1 pack-year increment | 0.999 (0.995-1.004) | 0.76 | 1.004 (0.999-1.009) | 0.14 | 1.003 (0.998-1.008) | 0.23 | 1.002 (0.997-1.008) | 0.43 |
| BA | Per 1 pack-year increment | 0.992 (0.983-1.001) | 0.07 | 0.994 (0.985-1.003) | 0.18 | 0.994 (0.985-1.003) | 0.18 | 0.992 (0.982-1.002) | 0.13 |

Abbreviations: ICAS indicates intracranial atherosclerotic stenosis; ECAS extracranial atherosclerotic stenosis; OR odds ration; Intra-ICA intracranial internal carotid artery; MCA middle cerebral artery; ACA anterior cerebral artery; PCA posterior cerebral artery; BA basilar artery.

OR_1_ adjusted for demographics (age and gender). OR_2_ adjusted for demographics (age and gender) and stroke risk factors (diabetes mellitus, hypertension, dyslipidemia, family history of stroke, history of cerebral ischemia, history of hemorrhagic stroke, heart disease). OR_3_ adjusted for all potential confounders including demographics, (age and gender), stroke risk factors (diabetes mellitus, hypertension, dyslipidemia, family history of stroke, history of cerebral ischemia, history of hemorrhagic stroke, heart disease), comorbidities (COPD, hepatic cirrhosis, peptic ulcer or previous GIB, arthritis, dementia, cancer and peripheral angiopathy), admission NIHSS, admission SBP and DBP, and admission blood tests (fasting blood glucose, TG, TC, HDL and LDL).

**Appendix A: The CICAS investigators**

Yognjun Wang, Beijing Tongren Hospital of Capital Medical University, Beijing; Xiaojun Zhang, Beijing Tongren Hospital of Capital Medical University, Beijing; Xiaojiang Sun, Shanghai Sixth People’s Hospital, Shanghai; Lan Yu, Tianjin Huanhu Hosptial, Tianjin; Minxia Guo, Shanxi Provincial People’s Hospital, Xi’an, Shanxi; Qilin Ma, The First Affiliated Hospital of Xiamen University, Xiamen, Fujian; Bo Xiao and Le Zhang, Xiangya Hospital Central South University, Changsha, Hunan; Zhong Zhang, No. 3 People’s Hospital of Chengdu, Chengdu, Sichuan; Anding Xu, The First Affiliated Hospital of Jinan University (Guangzhou Overseas Hospital), Guangzhou, Guangdong; Juntao Li, Handan Central Hospital, Handan, Heibei; Jie Lin, Handan First People’s Hospital, Handan, Heibei; Chengming Xing, Qingdao Municipal Hospital, Qingdao, Shandong; Yuming Xu, The First Affiliated Hospital of Zhengzhou University, Zhengzhou, Henan; Rongyuan Zheng and Zhao Han, The First Affiliated Hospital of Wenzhou Medical College (the First Provincial Wenzhou Hospital of Zhejiang), Wenzhou, Zhejiang; Xiaodong Yuan, The Affiliated Kailuan Hospital, North China Coal Medical College, Tangshan, Hebei; Wanlin Cui and Yuan Zou, The First Affiliated Hospital of Beifang Medical College, Zhangjiakou, Hebei; and Heli Yan, Shijiazhuang Center Hospital, Shijiazhuang, Hebei.

**Appendix B: Institutional review board within the CICAS network**

Institutional review board at Beijing Tiantan Hospital of Capital Medical University, Beijing;; Institutional review board at Beijing Tongren Hospital of Capital Medical University, Beijing; Institutional review board at Shanghai Sixth People’s Hospital, Shanghai; Institutional review board at Tianjin Huanhu Hosptial, Tianjin; Institutional review board at Shanxi Provincial People’s Hospital, Xi’an, Shanxi; Institutional review board at The First Affiliated Hospital of Xiamen University, Xiamen, Fujian; Institutional review board at Xiangya Hospital Central South University, Changsha, Hunan; Institutional review board at No. 3 People’s Hospital of Chengdu, Chengdu, Sichuan; Institutional review board at The First Affiliated Hospital of Jinan University (Guangzhou Overseas Hospital), Guangzhou, Guangdong; Institutional review board at Handan Central Hospital, Handan, Heibei; Institutional review board at Handan First People’s Hospital, Handan, Heibei; Institutional review board at Qingdao Municipal Hospital, Qingdao, Shandong; Institutional review board at The First Affiliated Hospital of Zhengzhou University, Zhengzhou, Henan; Institutional review board at The First Affiliated Hospital of Wenzhou Medical College (the First Provincial Wenzhou Hospital of Zhejiang), Wenzhou, Zhejiang; Institutional review board at The Affiliated Kailuan Hospital, North China Coal Medical College, Tangshan, Hebei; Institutional review board at The First Affiliated Hospital of Beifang Medical College, Zhangjiakou, Hebei; and Institutional review board at Shijiazhuang Center Hospital, Shijiazhuang, Hebei.
